# Supplementary material for: Umbilical Cord Plasma Lysophospholipids and Triacylglycerols Associated with Birthweight Percentiles
Source: Nutrients. 2024 Jan 17;16(2):274. doi: 10.3390/nu16020274 (PMC10820643; doi:10.3390/nu16020274)

Supplementary Table S1. Cord blood lipid concentrations quantified by LCMS showing the range and quartiles of each lipid species expressed in μg/mL.

Lipid	Min	25%	50%	75%	Max
Cer d16:1/C20:0	0.0003	0.0008	0.0010	0.0012	0.0025
Cer d16:1/C22:0	0.0011	0.0017	0.0024	0.0030	0.0056
Cer d16:1/C23:0	0.0003	0.0005	0.0006	0.0008	0.0030
Cer d16:1/C24:0	0.0022	0.0036	0.0046	0.0058	0.0117
Cer d16:1/C26:1	0.0002	0.0011	0.0014	0.0017	0.0035
Cer d17:0/C17:1	0.0010	0.0020	0.0024	0.0030	0.0058
Cer d17:0/C18:0	0.0006	0.0011	0.0013	0.0015	0.0027
Cer d17:0/C19:1	0.0004	0.0006	0.0009	0.0012	0.0106
Cer d17:0/C23:1	0.0017	0.0034	0.0042	0.0049	0.0101
Cer d17:0/C25:1	0.0098	0.0153	0.0189	0.0227	0.0428
Cer d17:0/C25:2	0.0025	0.0050	0.0059	0.0078	0.0139
Cer d17:1/C22:0	0.0007	0.0012	0.0015	0.0018	0.0029
Cer d17:1/C24:0	0.0026	0.0038	0.0044	0.0052	0.0117
Cer d18:0/C16:0	0.0006	0.0009	0.0012	0.0015	0.0036
Cer d18:0/C20:0	0.0005	0.0011	0.0013	0.0017	0.0026
Cer d18:0/C22:0	0.0028	0.0050	0.0058	0.0073	0.0154
Cer d18:0/C24:0	0.0069	0.0112	0.0129	0.0170	0.0362
Cer d18:0/C24:1	0.0035	0.0064	0.0084	0.0102	0.0185
Cer d18:0/C24:2	0.0003	0.0010	0.0012	0.0015	0.0025
Cer d18:0/C25:0	0.0002	0.0004	0.0007	0.0011	0.0038
Cer d18:0/C26:1	0.0003	0.0008	0.0010	0.0012	0.0021
Cer d18:1/C15:2	0.0006	0.0013	0.0016	0.0019	0.0050
Cer d18:1/C16:0	0.0146	0.0207	0.0235	0.0280	0.0509
Cer d18:1/C18:0	0.0047	0.0069	0.0089	0.0108	0.1267
Cer d18:1/C20:0	0.0038	0.0058	0.0069	0.0084	0.0387
Cer d18:1/C21:2	0.0012	0.0019	0.0024	0.0029	0.0118
Cer d18:1/C22:0	0.0198	0.0331	0.0411	0.0479	0.1081

Cer d18:1/C22:1	0.0003	0.0006	0.0008	0.0010	0.0086
Cer d18:1/C24:0	0.0869	0.1434	0.1791	0.2117	0.4147
Cer d18:1/C24:1	0.0277	0.0498	0.0596	0.0751	0.1235
Cer d18:1/C24:2	0.0030	0.0052	0.0064	0.0079	0.0150
Cer d18:1/C25:0	0.0031	0.0060	0.0099	0.0154	0.0294
Cer d18:1/C26:0	0.0030	0.0043	0.0049	0.0057	0.0163
Cer d18:1/C26:1	0.0013	0.0030	0.0033	0.0039	0.0078
Cer d18:1/C27:0	0.0032	0.0055	0.0070	0.0082	0.0135
Cer d18:1/C28:0	0.0003	0.0005	0.0005	0.0006	0.0053
Cer d18:1/C33:0	0.0011	0.0015	0.0017	0.0019	0.0049
Cer d18:1/C33:1	0.0008	0.0011	0.0012	0.0014	0.0108
Cer d18:2/C16:0	0.0111	0.0124	0.0136	0.0143	0.2187
Cer d18:2/C18:0	0.0007	0.0017	0.0021	0.0029	0.0065
Cer d18:2/C20:0	0.0006	0.0016	0.0022	0.0028	0.0046
Cer d18:2/C22:0	0.0047	0.0084	0.0105	0.0132	0.0220
Cer d18:2/C24:0	0.0149	0.0217	0.0291	0.0357	0.0576
Cer d18:2/C24:1	0.0058	0.0099	0.0124	0.0167	0.0301
Cer d19:0/C15:2	0.0113	0.0141	0.0149	0.0168	0.3752
Cer d19:0/C23:2	0.0085	0.0134	0.0148	0.0162	0.0203
Cer d19:0/C24:0	0.0002	0.0004	0.0006	0.0008	0.0020
Cer d19:0/C24:1	0.0003	0.0005	0.0006	0.0008	0.0019
Cer d19:0/C26:1	0.0005	0.0009	0.0010	0.0012	0.0023
Cer d20:1/C22:0	0.0009	0.0015	0.0018	0.0022	0.0042
Cer d20:1/C24:0	0.0027	0.0045	0.0055	0.0068	0.0106
Cer d20:1/C24:1	0.0013	0.0021	0.0026	0.0033	0.0055
Cer d20:1/C26:0	0.0009	0.0011	0.0012	0.0013	0.0066
DAG20:0	1.4106	1.9685	2.2182	2.4645	4.1750
DAG20:1	1.0516	1.3980	1.4845	1.6490	2.5705
DAG21:0	0.9083	1.1410	1.2173	1.3582	2.1538

DAG21:1	1.3412	2.0234	2.3680	2.8918	4.8949
DAG22:0	1.7604	2.4886	2.7618	3.0734	4.4179
DAG22:1	2.9373	3.9001	4.5498	5.2359	6.6115
DAG24:1	1.1843	1.6454	1.7867	2.0118	3.3364
DAG25:0	1.1369	1.4804	1.6491	1.8624	2.3678
DAG25:1	0.6843	0.8554	0.9599	1.0291	1.5411
DAG26:0	3.4491	4.7519	5.3208	6.0908	8.1178
DAG26:1	2.1404	2.6302	3.0043	3.4662	5.9615
DAG27:1	2.3824	3.4051	3.7770	4.1847	5.8817
DAG28:0	2.2402	2.9508	3.1937	3.5939	4.9630
DAG28:1	0.9738	1.2623	1.4308	1.5593	2.0887
DAG29:1	1.3074	1.6884	2.0350	2.4603	4.6735
DAG30:0	0.8889	1.1162	1.2632	1.3641	2.0558
DAG31:0	0.9820	1.2433	1.4123	1.5642	2.3805
DAG32:0	0.8530	1.1074	1.3438	1.4996	3.8940
DAG32:1	0.8843	1.4402	1.6917	1.9528	3.0178
DAG32:2	1.1313	1.3771	1.4902	1.6647	2.2518
DAG33:0	1.1917	1.4970	1.6810	1.9139	2.4731
DAG33:1	2.2739	3.4057	3.8431	4.4551	6.1959
DAG33:2	8.3653	15.8778	18.3672	22.1732	29.9807
DAG34:0	0.9938	1.2504	1.4060	1.5469	2.3652
DAG34:1	1.2157	1.6293	1.8175	2.0736	2.9695
DAG34:2	1.8109	2.5015	2.8177	3.1974	4.1081
DAG35:1	0.8925	1.1497	1.2480	1.4038	1.9761
DAG36:0	0.6856	0.8866	0.9895	1.0769	2.1814
DAG36:3	1.3782	1.8385	2.1657	2.4514	3.6260
DAG36:4	1.1570	1.5724	1.8171	1.9916	2.9572
DAG37:2	0.8908	1.1549	1.3502	1.5807	2.2994
DAG37:3	1.2904	1.8032	2.1730	2.4161	3.3955

DAG37:4	2.2592	2.7551	3.1138	3.5779	4.6716
DAG38:1	0.6873	0.8691	0.9634	1.0635	2.0171
DAG38:2	1.4413	1.7364	1.9252	2.0901	2.9670
DAG38:3	8.9172	12.0927	14.3834	16.5085	27.1713
DAG38:4	0.7740	1.0804	1.1772	1.3617	2.0868
DAG38:5	0.8831	1.0127	1.1515	1.3434	2.4312
DAG38:6	0.8169	1.0461	1.1973	1.3387	1.9179
DAG39:1	1.5573	1.8940	2.1610	2.3672	3.5200
DAG39:4	1.7638	2.4080	2.9542	3.4510	4.9019
DAG39:5	0.8928	1.0965	1.2465	1.4124	2.0395
DAG39:6	0.7809	0.9144	1.0675	1.2463	1.9118
DAG40:3	0.6672	0.8132	0.9155	1.0733	1.9813
DAG40:4	0.9142	1.2672	1.5208	1.6780	2.5658
DAG40:5	0.5991	0.7585	0.8629	0.9633	2.1983
DAG40:6	0.8149	1.1024	1.1900	1.3681	2.0650
DAG41:3	3.2198	5.1529	6.1615	6.9204	10.6000
DAG41:4	11.2337	16.5418	20.7512	23.3345	34.7549
DAG41:5	3.0799	4.0782	5.1275	6.3306	9.8267
DAG41:6	2.4344	3.2975	3.7157	4.0517	5.5681
DAG42:1	0.5759	0.7138	0.8303	0.9302	2.4602
DAG42:2	0.9667	1.2334	1.4315	1.5958	2.5136
DAG42:3	2.8639	3.8470	4.6561	5.2717	7.4745
DAG42:4	2.3374	3.4654	3.9867	4.4772	6.7481
DAG43:3	8.8609	13.5354	16.2080	18.7901	29.3124
DAG43:4	10.8440	15.8180	18.4765	21.3831	37.1257
DAG43:5	3.9014	5.7740	6.6935	7.9850	11.3895
DAG44:3	1.4229	2.1175	2.4786	2.7939	4.1464
DAG44:5	2.6084	3.5964	4.1937	4.7828	6.9451
DAG44:6	1.1620	1.6862	1.8197	2.1209	3.5938

DAG45:3	10.5759	14.9068	17.7374	20.6722	28.6577
DAG45:4	63.7659	82.9050	97.6362	112.6917	153.7407
DAG45:5	41.8876	58.8833	72.2297	84.7988	127.4412
DAG45:6	2.2097	3.4520	4.3540	5.4361	9.5349
DAG46:3	1.4290	1.9467	2.2993	2.6381	3.9303
DAG46:4	1.8518	2.6352	3.2800	3.8141	7.0607
DAG46:5	1.8739	2.6139	3.1197	3.5950	6.1815
DAG46:6	2.1157	3.1062	3.6867	4.1423	6.3578
DAG47:3	4.1895	6.1584	7.3758	8.5248	13.6786
DAG47:4	20.6158	29.5609	35.1299	42.0738	67.8169
DAG47:5	32.4059	44.6059	53.5065	63.2682	96.8193
DAG47:6	37.6723	55.1463	64.3747	71.1736	99.2249
DHCer d18:1/C16:0	0.0769	0.1012	0.1196	0.1486	0.2704
DHCer d18:1/C24:1	0.0057	0.0101	0.0126	0.0154	0.0294
LPC 14:0	0.0089	0.0117	0.0134	0.0153	0.0330
LPC 15:0	0.0036	0.0053	0.0060	0.0067	0.0107
LPC 16:0	1.1479	1.7882	1.9815	2.3199	3.0642
LPC 16:0p	0.0027	0.0035	0.0042	0.0048	0.0066
LPC 16:1	0.0456	0.0697	0.0829	0.1016	0.1680
LPC 17:1	0.0024	0.0031	0.0037	0.0045	0.0072
LPC 18:0	0.4892	0.8059	0.8896	1.0137	1.5269
LPC 18:1	0.3436	0.5659	0.6584	0.8332	1.1241
LPC 18:2	0.1814	0.3603	0.4541	0.5270	0.7908
LPC 18:3	0.0028	0.0049	0.0058	0.0066	0.0135
LPC 20:0p/20:1e	0.0009	0.0017	0.0019	0.0023	0.0034
LPC 20:1	0.0054	0.0077	0.0088	0.0105	0.0158
LPC 20:2	0.0070	0.0119	0.0148	0.0172	0.0267
LPC 20:3	0.0631	0.1044	0.1402	0.1709	0.3013
LPC 20:4	0.1495	0.3667	0.4410	0.5505	0.7463

LPC 20:5	0.0021	0.0054	0.0078	0.0096	0.0332
LPC 22:0	0.0018	0.0025	0.0029	0.0034	0.0074
LPC 22:1	0.0012	0.0016	0.0018	0.0021	0.0031
LPC 22:5	0.0066	0.0122	0.0142	0.0178	0.0252
LPC 22:6	0.0206	0.0502	0.0632	0.0802	0.1513
LPC 24:0	0.0074	0.0105	0.0118	0.0134	0.0182
LPE 16:0	0.1281	0.1906	0.2135	0.2614	0.3462
LPE 18:0	0.1320	0.2090	0.2380	0.2808	0.7961
LPE 18:1	0.0730	0.1215	0.1407	0.1719	0.2798
LPE 18:2	0.0493	0.1002	0.1194	0.1472	0.2441
MHCer d18:0/C14:0	0.0009	0.0015	0.0020	0.0025	0.0078
MHCer d18:1/C16:0	0.0595	0.0825	0.0972	0.1281	0.1808
MHCer d18:1/C18:0	0.0055	0.0097	0.0126	0.0177	0.0804
MHCer d18:1/C20:0	0.0043	0.0074	0.0094	0.0119	0.0452
MHCer d18:1/C22:0	0.0204	0.0350	0.0416	0.0536	0.1164
MHCer d18:1/C24:0	0.0489	0.0746	0.0943	0.1281	0.3208
MHCer d18:1/C24:1	0.0335	0.0497	0.0620	0.0795	0.1579
MHCer d18:2/C22:0	0.0031	0.0063	0.0084	0.0106	0.0220
oddPC 31:0	0.3545	0.4971	0.5862	0.6900	1.0327
oddPC 31:1	0.3333	0.4812	0.5476	0.6547	0.9913
oddPC 33:0	0.0694	0.1318	0.1500	0.1807	0.2886
oddPC 33:1	0.3889	0.5604	0.6924	0.8009	1.2213
oddPC 33:2	0.2166	0.3383	0.4096	0.4875	0.6652
oddPC 33:3	0.1784	0.2886	0.3302	0.4107	0.6328
oddPC 35:1	0.1312	0.2206	0.2631	0.3122	0.4542
oddPC 35:2	0.0868	0.1259	0.1552	0.1814	0.2849
oddPC 35:3	0.3624	0.6071	0.6705	0.7977	1.0682
oddPC 35:4	1.0317	2.6939	3.0945	3.4382	4.7364
oddPC 35:5	0.1455	0.5120	0.5847	0.6887	0.8932

oddPC 37:4	0.3167	0.8751	1.0152	1.1978	1.8331
oddPC 37:5	0.5083	1.4168	1.5510	1.7986	2.4156
oddPC 37:6	0.1996	0.5668	0.6635	0.8133	1.0367
oddPC 39:5	0.0593	0.1458	0.1660	0.1849	0.2646
oddPC 39:6	0.0912	0.1880	0.2148	0.2471	0.3526
oddPC 39:7	0.0868	0.1606	0.1931	0.2220	0.2853
PC 30:0	0.2943	0.4510	0.5119	0.5650	0.7257
PC 30:1	0.9986	1.4223	1.7147	1.9385	2.6639
PC 32:0	1.1604	1.6729	1.9712	2.1901	3.2387
PC 32:1	0.8947	1.3848	1.6221	1.9436	2.6637
PC 32:2	0.1840	0.2642	0.3010	0.3565	0.5784
PC 32:3	0.0043	0.0066	0.0080	0.0098	0.0155
PC 34:0	1.7256	2.1881	2.3914	2.6304	3.0364
PC 34:1	8.6859	11.0508	11.8914	13.2850	15.0186
PC 34:2	7.2729	9.1497	10.1348	11.9696	15.6630
PC 34:3	0.1946	0.3042	0.3659	0.4454	0.7289
PC 34:4	0.0209	0.0390	0.0455	0.0557	0.0881
PC 36:0	0.0930	0.1445	0.1722	0.2034	0.2798
PC 36:1	1.5378	2.0990	2.4053	2.8045	3.5517
PC 36:2	3.3102	4.5421	5.1941	6.0134	8.1236
PC 36:3	5.6228	8.4494	9.2092	10.2791	13.1041
PC 36:4	6.5718	13.0164	14.3535	15.6922	18.2043
PC 36:5	0.1655	0.4119	0.5605	0.6886	2.1142
PC 38:1	0.1787	0.2714	0.3282	0.3996	0.5915
PC 38:2	0.4808	0.7345	0.8342	1.0085	1.3878
PC 38:3	3.1198	4.8800	5.5414	6.3796	8.2914
PC 38:4	3.5659	8.2569	9.7358	10.9443	14.0346
PC 38:5	1.3505	2.9715	3.2634	3.5871	4.7516
PC 38:6	3.1250	6.0613	7.0953	8.5563	11.7429

PC 40:0	0.0156	0.0257	0.0286	0.0339	0.0446
PC 40:1	0.0027	0.0040	0.0049	0.0053	0.0074
PC 40:2	0.0080	0.0124	0.0139	0.0163	0.0213
PC 40:4	0.0832	0.2809	0.3420	0.4072	0.6862
PC 40:5	0.1944	0.4269	0.5391	0.6158	1.1599
PC 40:6	1.0058	1.8686	2.3372	3.0331	4.9941
PC 40:7	0.1197	0.2689	0.3083	0.3457	0.5700
PC(O-32:0)	0.3545	0.4971	0.5862	0.6900	1.0327
PC(O-32:1)	0.3333	0.4812	0.5476	0.6547	0.9913
PC(O-32:2)	0.0476	0.0668	0.0818	0.0934	0.1370
PC(O-34:0)	0.0112	0.0171	0.0213	0.0268	0.0456
PC(O-34:1)	0.3999	0.5619	0.7080	0.8010	1.2203
PC(O-34:2)	0.2166	0.3383	0.4096	0.4875	0.6652
PC(O-34:3)	0.1784	0.2886	0.3302	0.4107	0.6328
PC(O-34:4)	0.0046	0.0076	0.0091	0.0123	0.0277
PC(O-35:4)	0.0209	0.0390	0.0455	0.0557	0.0881
PC(O-36:1)	0.1312	0.2173	0.2561	0.2987	0.4419
PC(O-36:2)	0.1094	0.1556	0.1863	0.2193	0.3350
PC(O-36:3)	0.3624	0.6071	0.6705	0.7977	1.0682
PC(O-36:4)	1.0317	2.6939	3.0945	3.4382	4.7364
PC(O-36:5)	0.3433	1.2113	1.3610	1.6206	2.1479
PC(O-38:4)	0.3167	0.8751	1.0152	1.1978	1.8331
PC(O-38:5)	0.5451	1.2241	1.4065	1.6062	2.6283
PC(O-40:5)	0.0380	0.1004	0.1152	0.1345	0.2049
PC(O-40:6)	0.0707	0.2080	0.2505	0.2783	0.4536
PC(O-40:7)	0.0964	0.1749	0.2086	0.2388	0.3033
PC(P-30:0)	0.0133	0.0210	0.0244	0.0287	0.0410
PC(P-32:0)	0.3820	0.5392	0.6181	0.7313	1.0814
PC(P-32:1)	0.0476	0.0668	0.0818	0.0934	0.1370

PC(P-34:1)	0.2924	0.4146	0.5001	0.5942	0.7828
PC(P-34:2)	0.1784	0.2886	0.3302	0.4107	0.6328
PC(P-34:3)	0.0046	0.0077	0.0093	0.0126	0.0277
PC(P-36:1)	0.1094	0.1556	0.1863	0.2193	0.3350
PC(P-36:2)	0.3624	0.6071	0.6705	0.7977	1.0682
PC(P-36:3)	1.0317	2.6939	3.0945	3.4382	4.7364
PC(P-36:4)	0.3433	1.2113	1.3610	1.6206	2.1479
PC(P-38:1)	0.0311	0.0520	0.0627	0.0768	0.1163
PC(P-38:2)	0.0266	0.0463	0.0559	0.0626	0.0850
PC(P-38:4)	0.4835	1.1964	1.3753	1.4944	2.2172
PC(P-38:5)	0.1996	0.5668	0.6635	0.8133	1.0367
PC(P-38:6)	0.1147	0.1511	0.1760	0.2048	0.2547
PC(P-40:6)	0.0964	0.1749	0.2086	0.2388	0.3033
PE 32:1	0.0005	0.0020	0.0024	0.0031	0.0100
PE 34:1	0.0053	0.0218	0.0266	0.0323	0.0765
PE 34:2	0.0056	0.0260	0.0314	0.0389	0.0666
PE 34:3	0.0003	0.0010	0.0014	0.0017	0.0030
PE 35:2	0.0204	0.0943	0.1005	0.1112	1.1768
PE 36:0	0.0016	0.0065	0.0087	0.0105	0.0146
PE 36:1	0.0024	0.0081	0.0098	0.0115	0.0276
PE 36:2	0.0079	0.0291	0.0343	0.0422	0.0805
PE 36:3	0.0080	0.0303	0.0335	0.0389	0.1174
PE 36:4	0.0309	0.1013	0.1189	0.1419	0.2401
PE 36:5	0.0009	0.0037	0.0047	0.0055	0.0175
PE 38:2	0.0006	0.0011	0.0014	0.0018	0.0038
PE 38:3	0.0143	0.0480	0.0547	0.0661	0.1278
PE 38:4	0.0539	0.1629	0.1866	0.2212	0.4088
PE 38:5	0.0123	0.0400	0.0468	0.0587	0.1363
PE 38:6	0.0887	0.2657	0.3064	0.4019	1.0842

PE 40:4	0.0009	0.0026	0.0036	0.0039	0.0162
PE 40:6	0.0266	0.0651	0.0782	0.1073	0.2914
PE 40:7	0.0053	0.0139	0.0166	0.0199	0.0321
PE(O-18:1/20:3)	0.0031	0.0074	0.0101	0.0129	0.0295
PE(O-18:2/18:2)	0.0024	0.0077	0.0102	0.0132	0.0255
PE(O-18:2/20:3)	0.0116	0.0435	0.0517	0.0604	0.2958
PE(O-18:2/22:5)	0.0040	0.0183	0.0235	0.0286	0.0361
PE(O-34:2)	0.0056	0.0270	0.0297	0.0347	0.7474
PE(O-36:5)	0.0101	0.0453	0.0498	0.0566	0.7319
PE(O-40:6)	0.0072	0.0306	0.0354	0.0440	0.3383
PE(P-16:0/18:1)	0.0055	0.0264	0.0287	0.0336	0.6672
PE(P-16:0/20:4)	0.0101	0.0435	0.0478	0.0529	0.7292
PE(P-16:0/22:5)	0.0029	0.0135	0.0172	0.0205	0.0311
PE(P-16:0/22:6)	0.0016	0.0052	0.0071	0.0086	0.0139
PE(P-18:0/20:4)	0.0134	0.0500	0.0575	0.0655	0.4081
PE(P-18:0/22:5)	0.0086	0.0312	0.0356	0.0438	0.3383
PE(P-18:0/22:6)	0.0034	0.0144	0.0174	0.0211	0.0285
PE(P-36:4)	0.0101	0.0435	0.0478	0.0529	0.7292
PI 34:0	0.0013	0.0065	0.0086	0.0110	0.0245
PI 34:1	0.0762	0.1423	0.1641	0.1983	0.3082
PI 36:1	0.0257	0.0464	0.0564	0.0707	0.1010
PI 36:2	0.1343	0.2717	0.3329	0.3925	0.5967
PI 36:4	0.3405	0.6436	0.8144	0.9718	1.8908
PI 38:2	0.0750	0.1343	0.1592	0.1821	0.2845
PI 38:3	1.0589	1.4935	1.6671	2.0128	2.9335
PI 38:4	1.8742	3.5354	4.0961	4.7025	6.9032
PI 38:5	0.1706	0.3631	0.4346	0.5206	1.0449
PI 38:6	0.0751	0.1341	0.1624	0.2157	0.4209
PI 40:5	0.0778	0.1300	0.1675	0.2171	0.4261

PI 40:6	0.1341	0.2716	0.3400	0.4139	0.6746
PS 36:1	0.0021	0.0054	0.0072	0.0094	0.1216
PS 38:3	0.0019	0.0038	0.0059	0.0089	0.0621
PS 38:4	0.0088	0.0190	0.0234	0.0323	0.2367
PS 40:5	0.0008	0.0029	0.0038	0.0064	0.0185
PS 40:6	0.0051	0.0100	0.0127	0.0187	0.0493
SM d18:0/16:0	0.9430	1.5378	1.6816	1.8226	2.2892
SM d18:0/18:0	0.7433	1.1189	1.3257	1.6224	2.1914
SM d18:1/16:0	5.5447	7.9772	9.4800	10.4993	14.2309
SM d18:1/16:1	1.0943	1.5070	1.7939	2.1108	2.7064
SM d18:1/18:0	1.3980	2.0819	2.3976	2.7764	4.1057
SM d18:1/18:1	1.1803	1.6949	1.9222	2.2995	3.4699
SM d18:1/18:2	0.0230	0.0331	0.0403	0.0464	0.0700
SM d18:1/20:0	6.1051	7.7086	8.6788	10.0084	12.8235
SM d18:1/20:1	0.4719	0.7258	0.8639	0.9660	1.3793
SM d18:1/22:0	3.5464	4.6135	5.3609	6.1750	8.2883
SM d18:1/24:0	0.8191	1.2892	1.5566	1.8774	2.7803
SM d18:1/24:1	2.4699	3.4985	4.1171	4.9063	6.6670
SM d18:1/26:0	0.0134	0.0195	0.0227	0.0275	0.0392
SM d18:1/26:1	0.0396	0.0664	0.0773	0.0878	0.1141
SM d19:0/20:0	0.0743	0.1236	0.1440	0.1689	0.2604
TAG31:0	0.1913	0.2456	0.2726	0.3102	0.4471
TAG31:1	0.1496	0.1859	0.2103	0.2411	0.3741
TAG31:2	0.1133	0.1467	0.1626	0.1889	0.3095
TAG32:0	0.1223	0.1697	0.2067	0.2419	0.3809
TAG32:1	0.1472	0.1946	0.2200	0.2535	0.3730
TAG33:0	0.1517	0.2035	0.2268	0.2695	0.4357
TAG34:0	0.1662	0.2055	0.2346	0.2665	0.4707
TAG34:1	0.1660	0.2041	0.2377	0.2603	0.3699

TAG35:0	0.9931	1.3588	1.5112	1.7357	2.3540
TAG36:0	0.2395	0.3082	0.3667	0.4120	0.5254
TAG36:1	0.2022	0.2628	0.2991	0.3443	0.4691
TAG37:0	2.2315	3.8932	4.5794	5.3107	7.4470
TAG37:1	0.6121	0.9825	1.2105	1.4150	2.1552
TAG38:0	0.1748	0.2104	0.2326	0.2764	0.3794
TAG38:1	0.2733	0.3561	0.3918	0.4290	0.7269
TAG39:0	0.1353	0.1742	0.1955	0.2151	0.3368
TAG39:1	0.1049	0.1368	0.1512	0.1708	0.2798
TAG40:0	0.3813	0.4526	0.5106	0.5708	0.9029
TAG40:1	0.4549	0.6198	0.7061	0.7759	1.2532
TAG41:0	0.0935	0.1182	0.1348	0.1538	0.2357
TAG41:1	0.0905	0.1163	0.1288	0.1504	0.2319
TAG42:0	0.1771	0.2246	0.2514	0.2804	0.4003
TAG42:1	0.2201	0.2620	0.2892	0.3279	0.5087
TAG43:0	0.0927	0.1153	0.1322	0.1514	0.2311
TAG43:1	0.0924	0.1215	0.1391	0.1654	0.2578
TAG44:0	0.1304	0.1572	0.1708	0.1935	0.2981
TAG44:1	0.1402	0.1849	0.2017	0.2291	0.3833
TAG44:2	0.1419	0.1788	0.2022	0.2224	0.3475
TAG45:1	0.1082	0.1346	0.1513	0.1685	0.2600
TAG45:2	0.0972	0.1214	0.1357	0.1559	0.2532
TAG46:0	0.1183	0.1535	0.1695	0.1887	0.3186
TAG46:1	0.1405	0.1709	0.1896	0.2073	0.3437
TAG46:2	0.1576	0.1890	0.2153	0.2446	0.3812
TAG46:3	0.1573	0.2013	0.2421	0.2950	0.5264
TAG47:1	0.1092	0.1391	0.1601	0.1798	0.2634
TAG47:2	0.0837	0.1099	0.1246	0.1410	0.2144
TAG48:0	0.1677	0.2050	0.2280	0.2746	0.4078

TAG48:1	0.2261	0.2989	0.3272	0.3875	0.6369
TAG48:2	0.3456	0.7220	0.8872	1.2575	3.4328
TAG48:3	0.2607	0.5149	0.7239	0.8905	2.8448
TAG49:1	0.4543	0.5242	0.5625	0.5939	0.7542
TAG49:2	0.1288	0.1671	0.1805	0.2124	0.3048
TAG49:3	0.1347	0.2164	0.2665	0.3274	0.8835
TAG50:1	0.0896	0.1157	0.1284	0.1481	0.2600
TAG50:2	0.1339	0.1976	0.2410	0.3062	0.7891
TAG50:3	0.9420	2.7233	3.6003	4.7117	12.5675
TAG50:4	1.4229	2.9921	3.8674	5.3197	18.5311
TAG51:0	0.0928	0.1268	0.1495	0.1640	0.3468
TAG51:1	0.1509	0.2836	0.3759	0.4636	1.4837
TAG51:2	0.1483	0.2316	0.2788	0.3409	1.0257
TAG51:3	0.0716	0.0962	0.1121	0.1323	0.3006
TAG52:0	0.4010	0.6249	0.7551	0.9268	2.0593
TAG52:1	1.7694	4.6470	6.1871	8.8792	19.8820
TAG52:2	3.6128	6.4763	7.8405	10.9808	27.7644
TAG52:3	0.9616	2.0159	2.6066	3.4701	10.3841
TAG52:4	0.1858	0.3051	0.4096	0.5273	1.4911
TAG53:0	0.1219	0.1795	0.2335	0.2770	0.7272
TAG53:1	0.1157	0.1729	0.2173	0.2704	0.7996
TAG53:2	0.1104	0.1411	0.1697	0.1882	0.4098
TAG53:3	0.0561	0.0829	0.0965	0.1254	0.2650
TAG54:0	0.2274	0.5893	0.8197	1.1277	2.6836
TAG54:1	0.9909	1.7907	2.2297	2.8606	8.3499
TAG54:2	0.5265	0.9004	1.2047	1.6060	3.8940
TAG54:3	0.1835	0.2719	0.3377	0.4552	1.1406
TAG54:5	0.1729	0.2173	0.2513	0.2788	0.4470
TAG55:0	0.0633	0.0944	0.1105	0.1222	0.2377

TAG55:1	0.0621	0.0761	0.0885	0.1039	0.1946
TAG55:2	0.0846	0.1185	0.1394	0.1716	0.4113
TAG55:3	0.1026	0.2081	0.2499	0.3570	1.0399
TAG56:0	0.1177	0.1724	0.2045	0.2479	0.5750
TAG56:1	0.0829	0.1060	0.1244	0.1480	0.2555
TAG56:2	0.0793	0.1021	0.1200	0.1355	0.2167
TAG56:3	0.1264	0.1604	0.1792	0.2021	0.3064
TAG57:0	0.0665	0.0917	0.1044	0.1207	0.1815
TAG57:1	0.0704	0.0987	0.1129	0.1336	0.2198
TAG57:2	0.0851	0.1324	0.1561	0.1941	0.4171
TAG57:3	0.1718	0.3366	0.4442	0.5841	1.3526
TAG58:1	0.1957	0.2943	0.3636	0.4547	1.0724
TAG58:2	0.1069	0.1452	0.1735	0.2098	0.4827
TAG58:3	0.0656	0.0882	0.0990	0.1139	0.2020
TAG58:4	0.0534	0.0671	0.0786	0.0863	0.1429
TAG58:5	0.0459	0.0622	0.0689	0.0774	0.1291
TAG58:6	0.0441	0.0594	0.0679	0.0779	0.1244
TAG59:3	0.0503	0.0636	0.0703	0.0815	0.1186
TAG59:4	0.0416	0.0502	0.0564	0.0667	0.1117
TAG59:5	0.0399	0.0534	0.0638	0.0737	0.1202
TAG59:6	0.0449	0.0602	0.0723	0.0868	0.1506
TAG60:3	0.1790	0.2131	0.2432	0.2734	0.4349
TAG60:4	0.1041	0.1375	0.1514	0.1709	0.2585
TAG60:5	0.0633	0.0903	0.1019	0.1198	0.1931
TAG60:6	0.0548	0.0674	0.0766	0.0880	0.1407
TAG61:3	0.0816	0.1036	0.1150	0.1258	0.2220
TAG61:4	0.1199	0.1528	0.1645	0.1896	0.3026
TAG61:5	0.1226	0.1698	0.1821	0.2048	0.3216
TAG61:6	0.1187	0.1541	0.1719	0.1953	0.2981

Supplementary Table S2. Cord blood lipids associated with birthweight categories: small-for-gestational-age (SGA; birthweight <10th percentile; n=14) and large-for-gestational-age (LGA; >90th percentile; n=16), each group compared with the appropriately-grown-for-gestational age (AGA; 10-90th percentile; n=45) group

	Association with SGA†					Association with LGA†				
Lipid (predictor)	OR††	Lipid LCL	Lipid UCL	Uncorrected p-value	FDR corrected p-value	OR††	Lipid LCL	Lipid UCL	Uncorrected p-value	FDR corrected p-value
LPC 18:1	0.27	0.07	0.72	**2.24E-02**	4.97E-01	2.07	0.95	4.91	7.68E-02	9.49E-01
LPC 18:2	0.44	0.18	0.96	5.03E-02	4.97E-01	3.04	1.23	9.13	**2.68E-02**	9.49E-01
LPC 18:3	0.69	0.29	1.62	3.98E-01	8.09E-01	6.45	2.04	28.54	**4.86E-03**	9.49E-01
LPC 20:0p/20:1e	0.45	0.19	0.93	**4.75E-02**	4.97E-01	1.74	0.83	3.91	1.54E-01	9.49E-01
LPC 20:1	0.41	0.17	0.89	**3.52E-02**	4.97E-01	1.20	0.59	2.46	6.13E-01	9.49E-01
LPC 20:2	0.24	0.07	0.65	**1.18E-02**	4.97E-01	3.43	1.27	11.70	**2.70E-02**	9.49E-01
LPC 20:3	0.53	0.20	1.20	1.45E-01	6.37E-01	3.43	1.39	10.44	**1.44E-02**	9.49E-01
LPC 20:4	0.78	0.37	1.58	4.88E-01	8.54E-01	2.92	1.25	7.99	**2.15E-02**	9.49E-01
LPC 22:5	0.97	0.43	2.23	9.37E-01	9.73E-01	4.15	1.67	13.63	**6.92E-03**	9.49E-01
LPC 22:6	0.94	0.46	1.93	8.55E-01	9.47E-01	2.45	1.12	6.27	**3.85E-02**	9.49E-01
LPE 18:1	0.19	0.05	0.52	**4.93E-03**	4.97E-01	1.23	0.59	2.62	5.81E-01	9.49E-01
LPE 18:2	0.34	0.13	0.76	**1.42E-02**	4.97E-01	1.57	0.76	3.64	2.45E-01	9.49E-01
PC 34:1	1.30	0.63	2.85	4.93E-01	8.54E-01	0.43	0.16	0.92	**4.78E-02**	9.49E-01
PC 38:4	2.90	1.19	9.91	**4.78E-02**	4.97E-01	1.11	0.60	2.17	7.49E-01	9.55E-01
PC 38:6	2.89	1.19	9.04	**3.76E-02**	4.97E-01	1.04	0.55	2.03	9.03E-01	9.80E-01
PC 40:4	2.98	1.23	9.62	**3.59E-02**	4.97E-01	1.02	0.51	2.12	9.67E-01	9.94E-01
PE 32:1	0.97	0.50	1.86	9.15E-01	9.66E-01	0.34	0.11	0.80	**2.74E-02**	9.49E-01
PE 34:1	0.74	0.33	1.51	4.32E-01	8.32E-01	0.28	0.09	0.70	**1.57E-02**	9.49E-01
PE 36:2	1.07	0.52	2.16	8.58E-01	9.47E-01	0.37	0.12	0.88	**4.33E-02**	9.49E-01
PE 40:6	2.50	1.14	6.74	**3.80E-02**	4.97E-01	0.79	0.33	1.85	5.78E-01	9.49E-01
PI 36:4	2.38	1.14	5.83	**3.30E-02**	4.97E-01	1.27	0.64	2.54	4.82E-01	9.49E-01

PI 38:4	2.45	1.14	6.66	**4.27E-02**	4.97E-01	1.08	0.55	2.17	8.32E-01	9.80E-01
PI 38:6	2.41	1.16	5.80	**2.76E-02**	4.97E-01	1.40	0.75	2.70	2.87E-01	9.49E-01
PI 40:6	2.44	1.10	6.55	**4.47E-02**	4.97E-01	1.58	0.80	3.44	2.10E-01	9.49E-01
TAG50:2	4.32	1.14	20.90	**4.28E-02**	4.97E-01	0.32	0.06	1.33	1.35E-01	9.49E-01
TAG50:4	3.33	1.13	12.04	**4.03E-02**	4.97E-01	0.31	0.07	1.08	9.14E-02	9.49E-01
TAG51:1	2.36	1.08	5.94	**4.33E-02**	4.97E-01	0.75	0.33	1.68	4.82E-01	9.49E-01
TAG52:2	3.24	1.43	9.01	**1.02E-02**	4.97E-01	0.45	0.17	1.02	7.31E-02	9.49E-01
TAG52:3	3.78	1.53	12.08	**9.37E-03**	4.97E-01	0.41	0.14	1.02	7.38E-02	9.49E-01
TAG52:4	2.39	1.09	6.04	**4.02E-02**	4.97E-01	0.49	0.19	1.13	1.13E-01	9.49E-01
TAG53:3	2.81	1.20	8.27	**3.06E-02**	4.97E-01	0.50	0.18	1.21	1.47E-01	9.49E-01
TAG54:0	2.74	1.24	6.78	**1.81E-02**	4.97E-01	0.75	0.34	1.58	4.64E-01	9.49E-01
TAG54:1	2.60	1.09	7.23	**4.34E-02**	4.97E-01	0.31	0.09	0.86	**3.81E-02**	9.49E-01
TAG54:2	2.30	1.05	5.78	5.17E-02	4.97E-01	0.34	0.11	0.81	**2.70E-02**	9.49E-01
TAG54:3	3.17	1.11	11.30	**4.56E-02**	4.97E-01	0.31	0.08	0.97	5.86E-02	9.49E-01
TAG55:3	3.49	1.43	11.31	**1.46E-02**	4.97E-01	0.68	0.28	1.57	3.80E-01	9.49E-01
TAG56:0	2.84	1.29	7.71	**1.88E-02**	4.97E-01	0.33	0.11	0.79	**2.24E-02**	9.49E-01
TAG56:1	1.97	0.93	4.57	8.69E-02	5.40E-01	0.37	0.13	0.86	**3.34E-02**	9.49E-01
TAG57:3	3.49	1.40	10.90	**1.42E-02**	4.97E-01	0.68	0.28	1.57	3.77E-01	9.49E-01
TAG58:1	4.28	1.57	15.28	**1.02E-02**	4.97E-01	0.53	0.18	1.42	2.23E-01	9.49E-01
TAG58:2	3.49	1.43	11.02	**1.40E-02**	4.97E-01	0.53	0.18	1.34	1.97E-01	9.49E-01
TAG58:3	3.08	1.35	9.13	**1.71E-02**	4.97E-01	0.52	0.20	1.20	1.36E-01	9.49E-01
Cer d17:0/C17:1	2.99	1.29	9.03	**2.38E-02**	4.97E-01	1.25	0.65	2.54	5.04E-01	9.49E-01
Cer d17:0/C23:1	2.21	1.08	5.35	**4.75E-02**	4.97E-01	0.80	0.37	1.70	5.60E-01	9.49E-01
Cer d17:0/C25:1	2.31	1.12	5.88	**4.32E-02**	4.97E-01	0.72	0.34	1.48	3.72E-01	9.49E-01
Cer d18:0/C24:0	2.16	1.07	5.14	**4.89E-02**	4.97E-01	0.84	0.41	1.67	6.27E-01	9.49E-01
Cer d18:1/C22:0	2.36	1.12	6.16	**4.44E-02**	4.97E-01	0.82	0.38	1.74	6.03E-01	9.49E-01

Cer d18:1/C24:0	2.53	1.19	6.80	**3.45E-02**	4.97E-01	0.76	0.36	1.57	4.58E-01	9.49E-01
Cer d18:2/C18:0	0.93	0.46	1.88	8.36E-01	9.38E-01	0.45	0.20	0.90	**3.61E-02**	9.49E-01
Cer d18:2/C24:0	2.27	1.09	5.55	**4.40E-02**	4.97E-01	0.70	0.34	1.37	3.02E-01	9.49E-01
Cer d20:1/C22:0	2.18	1.11	4.92	**3.61E-02**	4.97E-01	1.17	0.60	2.31	6.47E-01	9.49E-01
Cer d20:1/C24:0	2.36	1.13	5.96	**3.71E-02**	4.97E-01	1.05	0.52	2.12	8.91E-01	9.80E-01

† Compared with AGA, adjusted for maternal age, maternal BMI, parity, mode of delivery, assisted conception, fasting glucose, 2h post-load glucose, and insulin treatment.

†† OR: Odds ratio with AGA as the reference category. An OR more than one indicates a positive association while an OR less than one indicates a negative association.

Supplementary Table S3. Cord blood lipids significantly associated with birthweight after excluding cases treated with insulin (sensitivity analysis 1, n = 72)

	Association between lipid and standardized birthweight percentile †				Fasting glucose influence on standardized birthweight percentile †				2h glucose influence on standardized birthweight percentile †			
Lipid species (predictor)	Beta Coefficient (BW%/SD log_{10} lipid) ††	Lipid LCL	Lipid UCL	FDR corrected p-value	Beta Coefficient (BW%/ mmol/L) †††	Lipid LCL	Lipid UCL	FDR corrected p-value	Beta Coefficient (BW%/ mmol/L) †††	Lipid LCL	Lipid UCL	FDR corrected p-value
LPC 20:2	20.71	11.41	30.02	9.56E-03	10.59	-7.70	28.89	3.84E-01	-3.37	-11.18	4.44	7.97E-01
LPC 18:1	18.19	9.55	26.84	1.14E-02	9.22	-9.34	27.79	3.84E-01	-1.32	-9.01	6.36	8.12E-01
LPC 16:1	16.86	7.21	26.51	3.61E-02	11.95	-7.23	31.12	3.84E-01	-2.74	-10.99	5.52	7.97E-01
LPC 18:2	15.19	6.70	23.69	3.61E-02	11.40	-7.70	30.51	3.84E-01	-1.56	-9.57	6.45	7.97E-01
LPE 18:1	15.48	6.69	24.26	3.61E-02	4.09	-15.54	23.73	6.86E-01	3.54	-4.28	11.35	7.97E-01
LPC 20:3	14.36	5.19	23.53	6.10E-02	10.54	-8.96	30.03	3.84E-01	-0.97	-9.11	7.17	8.63E-01
LPC 18:3	13.61	3.44	23.77	1.23E-01	17.73	-2.61	38.07	3.84E-01	-2.75	-11.56	6.07	7.97E-01
LPC 22:5	12.93	3.72	22.14	1.02E-01	11.46	-8.28	31.21	3.84E-01	-0.31	-8.50	7.87	9.67E-01
LPC 20:1	12.71	4.33	21.10	7.14E-02	6.05	-13.87	25.96	5.58E-01	1.39	-6.56	9.34	8.12E-01
LPE 18:2	12.67	3.69	21.64	1.02E-01	7.44	-12.52	27.40	4.72E-01	0.99	-7.05	9.03	8.60E-01
LPC 20:0p/20:1e	12.94	5.16	20.72	4.57E-02	13.24	-6.11	32.58	3.84E-01	-0.46	-8.44	7.53	9.44E-01
LPC 20:4	11.72	2.87	20.57	1.27E-01	13.27	-6.62	33.17	3.84E-01	-0.20	-8.43	8.04	9.77E-01
TAG50:2	-25.60	-40.32	-10.89	3.61E-02	10.46	-8.75	29.66	3.84E-01	0.81	-7.01	8.63	8.76E-01
TAG50:4	-23.59	-35.45	-11.74	1.38E-02	11.12	-7.62	29.86	3.84E-01	1.28	-6.33	8.89	8.14E-01
TAG54:1	-19.15	-28.77	-9.53	1.38E-02	15.16	-3.65	33.98	3.84E-01	-0.22	-7.90	7.47	9.75E-01
TAG54:3	-18.57	-30.11	-7.04	4.99E-02	13.74	-5.72	33.19	3.84E-01	0.69	-7.23	8.61	9.00E-01
TAG50:3	-18.40	-28.99	-7.81	3.61E-02	12.37	-6.83	31.56	3.84E-01	0.88	-6.94	8.69	8.67E-01
TAG52:3	-17.66	-26.37	-8.95	1.38E-02	14.62	-4.10	33.34	3.84E-01	0.17	-7.46	7.80	9.77E-01
TAG52:2	-17.55	-25.63	-9.47	9.56E-03	11.82	-6.57	30.22	3.84E-01	0.07	-7.45	7.59	9.88E-01
TAG58:1	-16.86	-26.97	-6.75	4.57E-02	9.95	-9.39	29.30	3.84E-01	1.27	-6.57	9.12	8.18E-01
TAG56:0	-16.74	-24.33	-9.15	9.56E-03	14.26	-4.10	32.63	3.84E-01	0.90	-6.55	8.36	8.62E-01

TAG54:2	-15.76	-24.15	-7.37	2.46E-02	15.96	-3.11	35.04	3.84E-01	-0.32	-8.11	7.47	9.64E-01
TAG52:1	-14.97	-24.14	-5.81	4.86E-02	12.97	-6.42	32.36	3.84E-01	0.02	-7.93	7.98	9.95E-01
TAG58:2	-14.91	-24.09	-5.73	4.86E-02	10.20	-9.21	29.61	3.84E-01	1.68	-6.19	9.55	7.97E-01
TAG52:4	-14.53	-23.05	-6.00	4.11E-02	12.62	-6.64	31.87	3.84E-01	1.10	-6.73	8.93	8.41E-01
TAG53:0	-14.46	-24.73	-4.19	1.02E-01	11.83	-7.91	31.56	3.84E-01	0.22	-7.90	8.33	9.75E-01
TAG54:0	-13.59	-21.94	-5.23	4.86E-02	9.68	-9.75	29.10	3.84E-01	1.58	-6.29	9.45	7.97E-01
TAG53:1	-13.42	-22.27	-4.57	7.14E-02	11.58	-7.98	31.15	3.84E-01	1.10	-6.86	9.07	8.43E-01
TAG55:3	-13.22	-22.41	-4.03	9.99E-02	8.90	-10.88	28.68	3.95E-01	1.71	-6.27	9.70	7.97E-01
TAG57:3	-13.09	-22.54	-3.64	1.02E-01	11.05	-8.72	30.83	3.84E-01	1.09	-6.96	9.14	8.46E-01
TAG53:3	-12.89	-21.96	-3.82	1.02E-01	9.44	-10.34	29.22	3.84E-01	1.66	-6.35	9.66	7.97E-01
TAG48:3	-12.79	-21.31	-4.28	7.32E-02	11.84	-7.75	31.42	3.84E-01	1.68	-6.27	9.63	7.97E-01
TAG56:1	-12.26	-20.36	-4.17	7.14E-02	10.29	-9.30	29.88	3.84E-01	1.72	-6.23	9.66	7.97E-01
TAG48:2	-12.00	-20.66	-3.34	1.02E-01	10.94	-8.83	30.72	3.84E-01	1.88	-6.14	9.90	7.97E-01
TAG58:3	-11.58	-19.91	-3.24	1.02E-01	9.76	-10.05	29.58	3.84E-01	1.61	-6.41	9.64	7.97E-01
TAG52:0	-11.24	-19.75	-2.74	1.27E-01	9.31	-10.64	29.26	3.90E-01	1.84	-6.22	9.90	7.97E-01

† Standardized for sex and gestational age using a local population reference calculated using methods described by Mikolajczyk, et al., 2011. [13]

†† Standardized birthweight percentile change for each SD increase in z-score standardized log_{10} lipid concentration

††† Standardized birthweight percentile change per mmol/L increase in maternal glucose concentration (fasting and 2h) determined in a 75g oral glucose tolerance test conducted at ~26 weeks' gestation (2hG). Statistical significance set at $p < 0.05$. Abbreviations: FG, fasting glucose; 2hG, 2h post-load glucose; FDR, false discovery rate; LCL, lower confidence limit; UCL, upper confidence limit.

Supplementary Table S4. Cord blood lipids significantly associated with birthweight, after excluding cases of diabetes in pregnancy defined by either a fasting glucose of ≥7.0 and/or 2h glucose ≥11.1 mmol/L in a mid-gestation 75g OGTT (sensitivity analysis 2, n = 72)

	Association between lipid and standardized birthweight percentile †				Fasting glucose influence on standardized birthweight percentile †				2h glucose influence on standardized birthweight percentile †			
**Lipid species (predictor)**	**Beta Coefficient (BW%/SD log_{10} lipid) ††**	**Lipid LCL**	**Lipid UCL**	**FDR corrected p-value**	**Beta Coefficient (BW%/ mmol/L) †††**	**Lipid LCL**	**Lipid UCL**	**FDR corrected p-value**	**Beta Coefficient (BW%/ mmol/L) †††**	**Lipid LCL**	**Lipid UCL**	**FDR corrected p-value**
LPC 20:2	21.81	12.32	31.29	6.76E-03	37.20	-5.57	79.97	0.57	-10.58	-24.58	3.42	1.00
LPC 18:1	18.55	9.80	27.30	9.09E-03	24.73	-18.42	67.89	0.57	-5.05	-18.78	8.68	1.00
LPC 16:1	18.02	8.15	27.89	2.76E-02	18.03	-26.48	62.54	0.57	-3.17	-17.21	10.88	1.00
LPC 18:2	16.05	7.39	24.71	2.58E-02	30.29	-14.18	74.77	0.57	-5.83	-20.05	8.39	1.00
LPE 18:1	15.58	6.55	24.60	3.40E-02	17.39	-27.58	62.35	0.57	-0.50	-14.60	13.60	1.00
LPC 20:3	14.91	5.62	24.20	5.18E-02	27.33	-18.08	72.74	0.57	-5.10	-19.63	9.43	1.00
LPC 18:3	14.34	4.33	24.35	8.19E-02	23.34	-22.65	69.33	0.57	-3.47	-18.07	11.13	1.00
LPC 22:5	13.24	4.17	22.30	7.49E-02	17.38	-28.67	63.43	0.57	-1.72	-16.17	12.73	1.00
LPC 20:1	12.65	4.08	21.21	7.08E-02	24.84	-20.99	70.68	0.57	-3.62	-18.17	10.94	1.00
LPE 18:2	12.77	3.54	21.99	1.00E-01	27.29	-18.98	73.55	0.57	-4.55	-19.34	10.25	1.00
LPC 20:0p/20:1e	12.44	4.48	20.40	5.72E-02	21.06	-24.45	66.58	0.57	-2.42	-16.77	11.94	1.00
LPC 20:4	12.12	3.33	20.92	1.00E-01	22.62	-23.57	68.81	0.57	-2.44	-17.02	12.15	1.00
TAG50:2	-26.28	-40.80	-11.77	2.76E-02	13.08	-31.76	57.91	0.61	0.94	-13.08	14.96	1.00
TAG50:4	-24.17	-36.03	-12.31	1.02E-02	10.30	-33.66	54.26	0.66	2.21	-11.54	15.96	1.00
TAG54:1	-19.86	-29.41	-10.31	9.09E-03	4.36	-39.89	48.61	0.85	3.62	-10.15	17.40	1.00
TAG54:3	-19.67	-31.37	-7.96	3.92E-02	4.93	-41.37	51.24	0.84	3.56	-10.82	17.93	1.00
TAG50:3	-18.67	-29.12	-8.23	2.91E-02	18.25	-26.42	62.92	0.57	-0.43	-14.45	13.59	1.00
TAG52:3	-18.22	-26.88	-9.55	9.09E-03	8.74	-35.02	52.50	0.71	2.71	-10.98	16.39	1.00
TAG52:2	-17.75	-25.72	-9.78	6.76E-03	7.87	-35.34	51.08	0.73	1.77	-11.69	15.23	1.00
TAG56:0	-17.60	-25.30	-9.89	6.76E-03	0.73	-42.79	44.25	0.97	5.10	-8.47	18.67	1.00

TAG58:1	-17.38	-27.42	-7.34	3.40E-02	12.95	-32.25	58.16	0.61	1.25	-12.89	15.38	1.00
TAG54:2	-16.69	-25.18	-8.21	1.41E-02	3.40	-41.55	48.34	0.88	3.77	-10.19	17.73	1.00
TAG58:2	-15.36	-24.55	-6.17	3.92E-02	12.84	-32.64	58.33	0.62	1.63	-12.61	15.86	1.00
TAG52:1	-15.35	-24.40	-6.29	3.82E-02	8.76	-36.99	54.52	0.72	1.58	-12.62	15.78	1.00
TAG52:4	-15.05	-23.68	-6.42	3.40E-02	11.27	-34.01	56.55	0.65	2.15	-12.02	16.32	1.00
TAG53:0	-14.98	-24.92	-5.04	6.93E-02	14.23	-31.85	60.32	0.60	0.50	-13.90	14.89	1.00
TAG53:1	-14.02	-22.82	-5.23	5.22E-02	15.04	-30.61	60.69	0.58	1.10	-13.21	15.41	1.00
TAG54:0	-13.92	-22.30	-5.54	3.93E-02	20.24	-24.90	65.38	0.57	-0.95	-15.14	13.23	1.00
TAG55:3	-13.74	-22.66	-4.83	6.17E-02	16.10	-29.70	61.90	0.57	0.67	-13.68	15.03	1.00
TAG57:3	-13.69	-22.95	-4.44	7.08E-02	20.33	-25.52	66.18	0.57	-0.67	-15.08	13.74	1.00
TAG53:3	-13.41	-22.41	-4.42	7.08E-02	12.23	-34.12	58.57	0.64	1.67	-12.81	16.14	1.00
TAG48:3	-13.06	-21.65	-4.47	6.71E-02	17.27	-28.56	63.09	0.57	0.62	-13.76	15.00	1.00
TAG56:1	-12.76	-20.85	-4.66	5.54E-02	5.80	-40.95	52.55	0.82	3.37	-11.14	17.88	1.00
TAG48:2	-12.28	-21.01	-3.55	9.16E-02	21.23	-24.87	67.34	0.57	-0.71	-15.20	13.78	1.00
TAG58:3	-11.96	-20.18	-3.74	7.49E-02	13.69	-32.67	60.05	0.61	1.15	-13.34	15.63	1.00
TAG52:0	-11.61	-20.06	-3.15	1.00E-01	15.30	-31.26	61.85	0.58	0.62	-13.93	15.18	1.00

† Standardized for sex and gestational age using a local population reference calculated using methods described by Mikolajczyk, et al., 2011 [13]

†† Standardized birthweight percentile change for each SD increase in z-score standardized $\log_{10}$ lipid concentration

††† Standardized birthweight percentile change per mmol/L increase in maternal glucose concentration (fasting and 2h) determined in a 75g oral glucose tolerance test conducted at ~26 weeks' gestation (2hG). Abbreviations: FG, fasting glucose; 2hG, 2h post-load glucose; FDR, false discovery rate; LCL, lower confidence limit; UCL, upper confidence limit.

Supplementary Table S5. Cord blood lipids significantly associated with birthweight after excluding cases of possible placental insufficiency or hypertensive disorders of pregnancy (sensitivity analysis 3, n = 55)

	Association between lipid and standardized birthweight percentile †				Fasting glucose influence on standardized birthweight percentile †				2h glucose influence on standardized birthweight percentile †			
**Lipid species (predictor)**	**Beta Coefficient (BW%/SD log_{10} lipid) ††**	**Lipid LCL**	**Lipid UCL**	**FDR corrected p-value**	**Beta Coefficient (BW%/ mmol/L) †††**	**Lipid LCL**	**Lipid UCL**	**FDR corrected p-value**	**Beta Coefficient (BW%/ mmol/L) †††**	**Lipid LCL**	**Lipid UCL**	**FDR corrected p-value**
LPC 20:2	17.17	8.15	26.18	7.00E-02	6.42	-11.18	24.03	0.56	-6.18	-14.84	2.49	0.50
LPC 18:1	14.58	6.65	22.52	7.00E-02	4.74	-13.10	22.58	0.62	-4.02	-12.69	4.66	0.50
LPC 16:1	15.09	5.96	24.22	1.51E-01	3.72	-14.64	22.08	0.70	-3.90	-12.78	4.97	0.50
LPC 18:2	15.80	7.17	24.44	7.00E-02	4.16	-13.74	22.06	0.66	-4.13	-12.81	4.55	0.50
LPE 18:1	8.35	-0.61	17.31	5.79E-01	6.23	-13.38	25.85	0.57	-2.60	-12.27	7.07	0.60
LPC 20:3	12.84	4.49	21.18	1.84E-01	6.83	-11.56	25.22	0.56	-5.42	-14.44	3.60	0.50
LPC 18:3	12.54	4.17	20.90	1.84E-01	12.33	-6.26	30.91	0.56	-6.57	-15.74	2.59	0.50
LPC 22:5	13.69	6.32	21.06	7.00E-02	8.88	-8.79	26.55	0.56	-5.66	-14.35	3.03	0.50
LPC 20:1	10.05	1.42	18.69	3.21E-01	3.11	-16.55	22.76	0.76	-3.35	-12.71	6.01	0.50
LPE 18:2	9.44	1.01	17.87	3.39E-01	6.83	-12.37	26.04	0.56	-4.26	-13.63	5.11	0.50
LPC 20:0p/20:1e	10.83	2.93	18.73	1.95E-01	9.76	-8.94	28.46	0.56	-5.22	-14.40	3.96	0.50
LPC 20:4	10.80	3.09	18.51	1.87E-01	9.38	-9.25	28.00	0.56	-5.10	-14.24	4.05	0.50
TAG50:2	-19.91	-33.36	-6.46	1.84E-01	6.81	-11.70	25.33	0.56	-4.14	-13.18	4.91	0.50
TAG50:4	-20.48	-31.71	-9.25	7.00E-02	7.24	-10.53	25.00	0.56	-3.27	-11.97	5.43	0.50
TAG54:3	-14.88	-26.59	-3.17	2.38E-01	9.73	-9.15	28.62	0.56	-4.31	-13.55	4.93	0.50
TAG50:3	-14.36	-24.27	-4.45	1.84E-01	8.54	-9.99	27.07	0.56	-3.93	-13.01	5.15	0.50
TAG54:1	-13.43	-22.99	-3.86	1.87E-01	9.44	-9.18	28.07	0.56	-4.27	-13.39	4.84	0.50
TAG52:3	-12.20	-20.99	-3.42	1.87E-01	8.95	-9.69	27.60	0.56	-3.71	-12.85	5.42	0.50
TAG58:1	-11.41	-21.96	-0.86	3.64E-01	5.99	-13.38	25.35	0.58	-3.28	-12.71	6.16	0.51
TAG52:2	-11.65	-19.62	-3.68	1.84E-01	8.09	-10.42	26.60	0.56	-4.26	-13.32	4.80	0.50
TAG56:0	-13.20	-21.30	-5.09	1.51E-01	10.09	-8.09	28.28	0.56	-4.36	-13.25	4.54	0.50

TAG54:2	-11.39	-19.53	-3.25	1.87E-01	10.21	-8.45	28.87	0.56	-4.30	-13.43	4.82	0.50
TAG58:2	-11.27	-21.15	-1.40	3.21E-01	6.51	-12.68	25.71	0.56	-3.64	-13.00	5.73	0.50
TAG52:1	-10.25	-18.35	-2.15	2.38E-01	8.92	-9.96	27.80	0.56	-4.35	-13.59	4.90	0.50
TAG52:4	-12.65	-21.09	-4.21	1.84E-01	7.88	-10.55	26.32	0.56	-3.30	-12.34	5.75	0.50
TAG53:0	-12.49	-22.48	-2.49	2.48E-01	6.59	-12.39	25.58	0.56	-4.35	-13.61	4.91	0.50
TAG54:0	-9.92	-18.19	-1.65	2.85E-01	6.72	-12.35	25.78	0.56	-3.51	-12.83	5.81	0.50
TAG53:1	-11.96	-21.36	-2.57	2.38E-01	6.37	-12.59	25.32	0.56	-3.45	-12.70	5.81	0.50
TAG55:3	-9.72	-18.27	-1.17	3.21E-01	5.59	-13.71	24.89	0.60	-3.42	-12.80	5.96	0.50
TAG57:3	-8.40	-17.60	0.79	5.97E-01	7.01	-12.53	26.56	0.56	-3.80	-13.34	5.74	0.50
TAG53:3	-11.36	-20.24	-2.49	2.38E-01	6.19	-12.76	25.15	0.57	-4.30	-13.53	4.93	0.50
TAG48:3	-11.68	-19.97	-3.38	1.87E-01	8.70	-9.91	27.31	0.56	-3.66	-12.78	5.46	0.50
TAG48:2	-12.91	-21.14	-4.68	1.82E-01	8.16	-10.13	26.45	0.56	-3.96	-12.91	5.00	0.50
TAG52:0	-10.51	-18.73	-2.30	2.38E-01	6.26	-12.69	25.21	0.57	-3.92	-13.16	5.31	0.50
TAG58:3	-6.37	-14.98	2.24	8.08E-01	7.74	-12.00	27.47	0.56	-4.50	-14.16	5.15	0.50
TAG56:1	-9.00	-16.83	-1.17	3.21E-01	7.79	-11.31	26.89	0.56	-4.93	-14.30	4.44	0.50

† Standardized for sex and gestational age using a local population reference calculated using methods described by Mikolajczyk, et al., 2011 [13]

†† Standardized birthweight percentile change for each SD increase in z-score standardized log_{10} lipid concentration

††† Standardized birthweight percentile change per mmol/L increase in maternal glucose concentration (fasting and 2h) determined in a 75g oral glucose tolerance test conducted at ~26 weeks' gestation (2hG). Abbreviations: FG, fasting glucose; 2hG, 2h post-load glucose; FDR, false discovery rate; LCL, lower confidence limit; UCL, upper confidence limit.

Supplementary Table S6. Cord blood lipids associated with birthweight and the relative influence of the covariates of fasting and 2h maternal glucose (Unadjusted model).

	**Association between lipid and standardized birthweight percentile †**				**Fasting glucose influence on standardized birthweight percentile †**				**2h glucose influence on standardized birthweight percentile †**			
**Lipid species (predictor)**	**Beta Coefficient (BW%/SD log_{10} lipid) ††**	**Lipid LCL**	**Lipid UCL**	**FDR corrected p-value**	**Beta Coefficient (BW%/ mmol/L) †††**	**Lipid LCL**	**Lipid UCL**	**FDR corrected p-value**	**Beta Coefficient (BW%/ mmol/L) †††**	**Lipid LCL**	**Lipid UCL**	**FDR corrected p-value**
**LPC 20:2**	18.11	10.45	25.77	3.16E-03	8.69	-8.93	26.32	5.90E-01	-0.78	-7.50	5.94	8.67E-01
**TAG52:2**	-16.25	-23.14	-9.36	3.16E-03	8.19	-9.44	25.83	5.90E-01	0.55	-6.11	7.20	9.13E-01
**LPC 18:1**	16.74	9.49	23.98	3.16E-03	7.58	-10.15	25.30	5.90E-01	-0.02	-6.73	6.69	9.95E-01
**TAG50:4**	-22.45	-32.82	-12.08	4.61E-03	8.00	-9.97	25.97	5.90E-01	1.15	-5.61	7.92	8.43E-01
**TAG56:0**	-15.00	-22.02	-7.98	4.61E-03	9.59	-8.43	27.61	5.90E-01	1.16	-5.62	7.94	8.43E-01
**TAG54:1**	-17.60	-25.79	-9.42	4.61E-03	11.25	-6.79	29.28	5.90E-01	0.39	-6.41	7.19	9.42E-01
**TAG52:3**	-16.48	-24.13	-8.83	4.61E-03	10.94	-7.08	28.96	5.90E-01	0.23	-6.57	7.04	9.66E-01
**LPC 18:2**	15.41	7.90	22.92	7.15E-03	8.75	-9.40	26.91	5.90E-01	-0.14	-7.03	6.76	9.84E-01
**LPE 18:1**	14.66	7.25	22.06	9.56E-03	2.55	-15.97	21.06	7.90E-01	4.42	-2.59	11.42	8.43E-01
**TAG54:2**	-14.29	-21.52	-7.06	9.56E-03	11.94	-6.42	30.31	5.90E-01	0.08	-6.85	7.01	9.88E-01
**TAG58:1**	-17.67	-26.75	-8.59	1.06E-02	6.34	-12.02	24.70	5.90E-01	1.63	-5.26	8.53	8.43E-01
**LPC 16:1**	15.26	7.34	23.17	1.07E-02	10.32	-8.06	28.70	5.90E-01	-1.62	-8.74	5.51	8.43E-01
**TAG50:2**	-23.48	-35.72	-11.25	1.07E-02	6.37	-12.03	24.77	5.90E-01	1.28	-5.64	8.19	8.43E-01
**TAG55:3**	-14.39	-22.01	-6.77	1.22E-02	5.29	-13.20	23.79	6.03E-01	1.81	-5.12	8.74	8.43E-01
**LPC 22:5**	13.84	6.36	21.33	1.45E-02	8.96	-9.52	27.44	5.90E-01	0.10	-6.92	7.11	9.88E-01
**TAG58:2**	-15.15	-23.54	-6.76	1.59E-02	6.53	-12.05	25.11	5.90E-01	1.67	-5.31	8.65	8.43E-01
**TAG50:3**	-16.71	-25.96	-7.47	1.59E-02	8.44	-10.10	26.99	5.90E-01	1.04	-5.95	8.03	8.46E-01
**LPC 20:3**	14.33	6.42	22.25	1.59E-02	7.93	-10.61	26.48	5.90E-01	0.35	-6.67	7.37	9.51E-01
**TAG52:4**	-13.33	-20.81	-5.84	1.77E-02	9.37	-9.22	27.96	5.90E-01	0.83	-6.19	7.84	8.67E-01
**TAG57:3**	-14.86	-23.34	-6.38	1.94E-02	7.56	-11.07	26.20	5.90E-01	1.26	-5.75	8.27	8.43E-01
**TAG53:3**	-13.83	-21.73	-5.93	1.94E-02	5.66	-13.04	24.36	6.01E-01	1.57	-5.44	8.58	8.43E-01
**TAG54:0**	-13.40	-21.16	-5.64	2.04E-02	5.57	-13.18	24.31	6.03E-01	1.83	-5.20	8.85	8.43E-01

**TAG52:1**	-12.79	-20.19	-5.39	2.04E-02	8.75	-9.92	27.41	5.90E-01	0.83	-6.21	7.87	8.67E-01
**LPE 18:2**	12.76	5.30	20.22	2.07E-02	5.24	-13.55	24.02	6.09E-01	2.46	-4.58	9.50	8.43E-01
**TAG53:1**	-12.75	-20.20	-5.31	2.07E-02	8.00	-10.69	26.69	5.90E-01	0.97	-6.08	8.01	8.47E-01
**LPC 18:3**	13.56	5.55	21.57	2.15E-02	15.41	-3.76	34.57	5.90E-01	-2.02	-9.41	5.37	8.43E-01
**TAG54:3**	-17.25	-27.44	-7.06	2.15E-02	10.06	-8.69	28.80	5.90E-01	0.68	-6.39	7.75	8.92E-01
**TAG53:0**	-13.05	-21.02	-5.08	2.79E-02	7.82	-10.98	26.63	5.90E-01	0.76	-6.34	7.86	8.77E-01
**LPC 20:0p/20:1e**	12.12	4.72	19.53	2.79E-02	10.33	-8.51	29.16	5.90E-01	0.24	-6.89	7.38	9.66E-01
**TAG48:3**	-12.70	-20.50	-4.90	2.83E-02	8.61	-10.20	27.42	5.90E-01	1.09	-5.99	8.18	8.46E-01
**TAG56:1**	-11.28	-18.61	-3.95	4.50E-02	5.66	-13.36	24.69	6.03E-01	1.72	-5.41	8.84	8.43E-01
**LPC 20:4**	12.09	4.24	19.94	4.50E-02	10.10	-8.87	29.07	5.90E-01	-0.04	-7.26	7.18	9.94E-01
**LPC 20:1**	11.65	3.99	19.32	4.84E-02	3.23	-16.04	22.51	7.47E-01	2.01	-5.13	9.15	8.43E-01

† Standardized for sex and gestational age using a local population reference calculated using methods described by Mikolajczyk, et al., 2011 [13]

†† Standardized birthweight percentile change for each SD increase in z-score standardized $\log_{10}$ lipid concentration

††† Standardized birthweight percentile change per mmol/L increase in maternal glucose concentration (fasting and 2h) determined in a 75g oral glucose tolerance test conducted at ~26 weeks' gestation (2hG). Abbreviations: FG, fasting glucose; 2hG, 2h post-load glucose; FDR, false discovery rate; LCL, lower confidence limit; UCL, upper confidence limit.

Supplementary Figure S1. Study participant flowchart

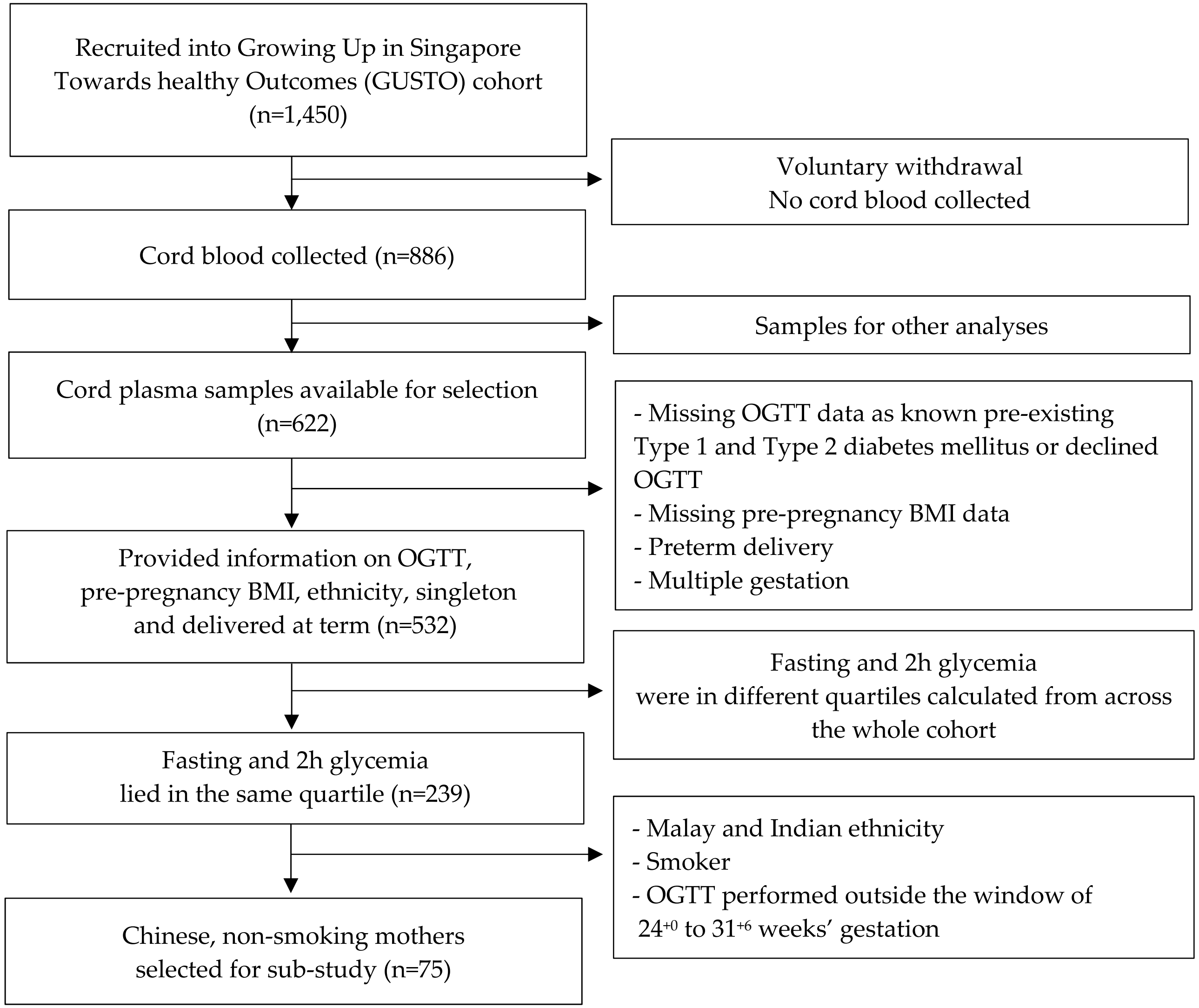

Supplement: Supplementary file 1 [file nutrients-16-00274-s001.zip › nutrients-2745607-supplementary.pdf]
